# Development and Fabrication of a Molecularly Imprinted Polymer-Based Electroanalytical Sensor for the Determination of Acyclovir

**DOI:** 10.1021/acsomega.3c09399

**Published:** 2024-02-12

**Authors:** Abdullah Al Faysal, Ahmet Cetinkaya, Sariye Irem Kaya, Taner Erdoğan, Sibel A. Ozkan, Ayşegül Gölcü

**Affiliations:** † Faculty of Sciences and Letters, Department of Chemistry, Istanbul Technical University, Maslak, Istanbul 34469, Turkey; ‡ Faculty of Pharmacy, Department of Analytical Chemistry, Ankara University, Ankara 06560, Turkey; § Graduate School of Health Sciences, Ankara University, Ankara 06110, Turkey; ∥ Gulhane Faculty of Pharmacy, Department of Analytical Chemistry, University of Health Sciences, Ankara 06018, Turkey; ⊥ Kocaeli Vocational School, Department of Chemistry and Chemical Processing Technologies, Kocaeli University, Kocaeli 41140, Turkey

## Abstract

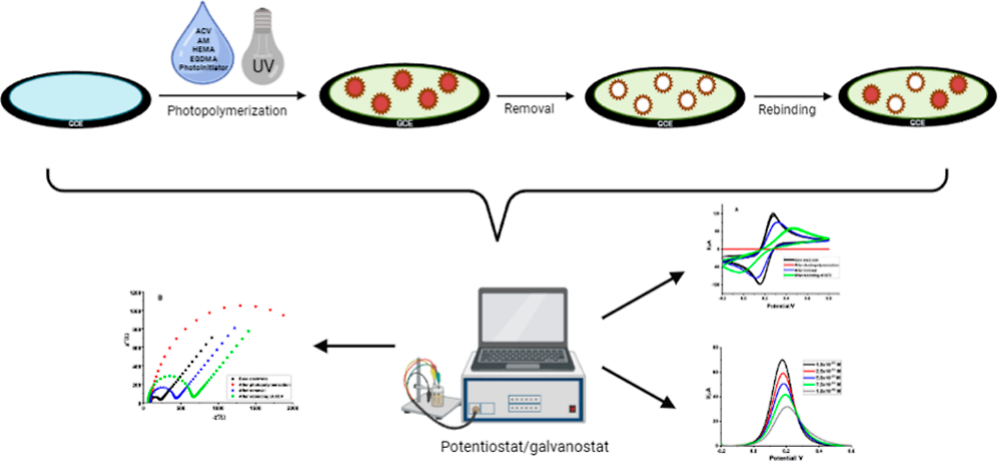

Acyclovir (ACV), a synthetic nucleoside derivative of purine, is one of the most potent antiviral medications recommended in the specific management of varicella-zoster and herpes simplex viruses. The molecularly imprinted polymer (MIP) was utilized to create an effective and specific electrochemical sensor using a straightforward photopolymerization process to determine ACV. The polymeric thin coating was developed using the template molecule ACV, a functional monomer acrylamide, a basic monomer 2-hydroxyethyl methacrylate, a cross-linker ethylene glycol dimethacrylate, and a photoinitiator 2-hydroxy-2-methyl propiophenone on the exterior of the glassy carbon electrode (GCE). Scanning electron microscopy, attenuated total reflectance–Fourier transform infrared spectroscopy, electrochemical impedance spectroscopy, and cyclic voltammetry were employed for the purpose of characterizing the constructed sensor (AM-ACV@MIP/GCE). Differential pulse voltammetry and a 5 mM ferrocyanide/ferricyanide ([Fe(CN)_6_]^3–/4–^) redox reagent were used to detect the ACV binding to the specific cavities on MIP. The study involves density functional theory (DFT) calculations, which were conducted to investigate template-functional monomer interactions thoroughly, calculate template-functional monomer interaction energies, and determine the optimal template/functional monomer ratio. DFT calculations were performed using Becke’s three-parameter hybrid functional with the Lee–Yang–Parr correlation functional (B3LYP) method and 6-31G(d,p) basis set. The sensor exhibits linear performance throughout the concentration region 1 × 10^–11^ to 1 × 10^–10^ M, and the limit of detection and limit of quantification were 7.15 × 10^–13^ M and 2.38 × 10^–12^ M, respectively. For the electrochemical study of ACV, the sensor demonstrated high accuracy, precision, robustness, and a short detection time. Furthermore, the developed electrochemical sensor exhibited exceptional recovery in tablet dosage form and commercial human blood samples, with recoveries of 99.40 and 100.44%, respectively. The findings showed that the AM-ACV@MIP/GCE sensor would effectively be used to directly assess pharmaceuticals from actual specimens and would particularly detect ACV compared to structurally similar pharmaceutical compounds.

## 1 Introduction

**Figure 1:**
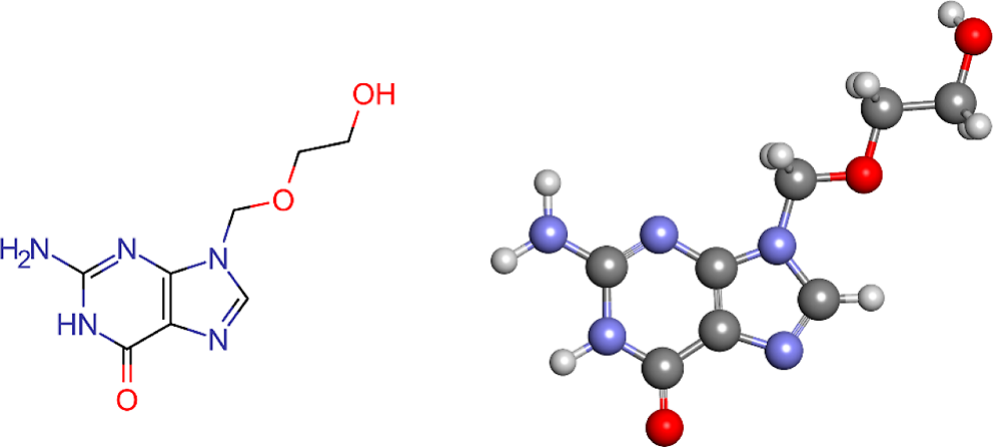
Molecular structure of ACV.

The majority of viruses that cause illness and occasionally mortality in people and other animals are pathogens that may damage structures such as cells, tissues, and organs.^1^ According to reports, two million people die from viral infections each year worldwide.^2^ Antiviral medications are utilized for the management of particular viral disorders, while a wide variety of antivirals are effective against several viruses.^3^ Acyclovir (ACV) (Figure 1), currently one of the world’s most frequently prescribed antiviral medications, is produced from a guanosine derivative.^4^ Because of its excellent specificity and minimal cytotoxicity, it is seen as the start of an entirely new phase in antiviral therapy.^5^ As the initial course of action and preventive therapy for herpes simplex virus infections, varicella-zoster, Epstein–Barr, herpes zoster, and acute herpetic keratitis viruses, ACV is the gold standard medication that doctors recommend.^4,6,7^ Because of low solubility in water,^8^ rapid half-life (3 h),^9^ low absorption through the skin,^8^ and limited oral bioavailability (15–30%),^10^ to obtain the required therapeutic effect, patients receive standard dosage forms of ACV frequently (5–6 times daily)^9^ as well as in substantial amounts (200–800 mg).^8^ In addition to causing nausea and diarrhea, high doses of ACV in humans can have other possibly dangerous adverse effects, like acute kidney disease.^11^ ACV levels must be identified and analyzed in commercial pharmaceutical preparations and human serum and urine samples because of the toxicological and adverse health effects to ensure that patients are using medications safely.

For the determination of ACV, a variety of analytical methods, including spectrophotometry,^12^ high-performance liquid chromatography,^13,14^ liquid chromatography/tandem mass spectrometry (LC–MS/MS),^15^ electrochemical analysis (square wave voltammetry),^16,17^ and flow injection-chemiluminescence (FI-CL),^18^ have been reported. These techniques are typically intricate, requiring expensive instruments, complicated operations, significant expenses for analysis, and laborious steps. They also frequently require specialized scientific instruments and staff with advanced training.^19,20^

Exceptional features of electrochemical techniques include great accuracy, short detection times, low expenses, ease of use of the instruments, and potential for downsizing and incorporating portable equipment.^21,22^ Consequently, the detection of minute concentrations of pharmaceuticals as well as different analytes in biological, pharmaceutical, and environmental samples is commonly accomplished through the use of electrochemical sensors.^23−26^ Even with these benefits, selectivity restrictions, the inability to use electrochemical sensors for anything other than electroactive compounds, and the possibility of electrode fouling remain problems for electrochemical sensors. Molecularly imprinted polymers (MIPs) have been used in recent decades in conjunction with electrochemical systems for the determination of a variety of molecules since they are capable of providing high affinity and specificity, improved stability, simple preparation, a variety of template options, and simplicity of adaptability to practical applications. It is theoretically possible to manufacture imprinted polymers for any desired analyte. As many as 10,000 chemicals and biological components including inorganic ions, pharmaceuticals, nucleic acids, proteins, and even organisms have all been efficiently imprinted.^27,28^ A plethora of publications detailing the implementation of MIPs in numerous drug assessments have been reported in recent years.^27,29,30^

Molecular imprinting is the method of inducing sites for identifying a particular molecule within the polymer substance by using a template and a functional monomer. This procedure involves polymerizing a combination of monomer materials surrounding a selected target that serves as a template. After removing the templates from the created polymer, binding regions are left behind that are specifically able to recognize the target molecules because they are identical to them in terms of shape, dimension, and functional groups.^31^ Bulk polymerization is often used to create MIPs. Unfortunately, there are a number of issues with this approach, including uneven binding site distribution, inadequate site accessibility, and insufficient removal of the imprinted template. The surface imprinting technology has been created as a solution to the above-specified issues.^32^ Thus, by employing the MIP approach, which uses ACV as a template and monomer acrylamide (AM) as the functional monomer, ACV was determined for the first time. In this study, AM is the functional monomer that interacts with ACV and has primary activity in polymerization. 2-Hydroxyethyl methacrylate (HEMA), as the basic monomer, is effective in the formation of the polymerization chain. The photopolymerization technique was utilized on the glassy carbon electrode (GCE) to create the MIP-based sensor (AM-ACV@MIP/GCE). The method used to initiate the polymerization of an MIP is a crucial factor to take into account while constructing an MIP since it influences the parameters of the reaction and, consequently, the properties of the resultant MIP. Currently, photopolymerization is a widely used method for MIPs. This technique has acquired significant momentum recently and is frequently the preferred strategy.^33^ Because UV light directly causes radical production, it safeguards heat-sensitive noncovalent connections among the functional monomers and templates.^34,35^

Several methods for determining ACV have been investigated in earlier research. However, these approaches are more expensive overall, require more time for analysis, and are unsuitable for routine testing. Furthermore, despite the fact that MIP-based electrochemical sensors are increasingly being used by researchers for drug analysis, none of these earlier investigations have looked into them. The suggested study aims to offer a novel, highly accurate, and specific MIP sensor developed on the exterior of a GCE for the quantification of ACV using differential pulse voltammetry (DPV). In this work, template-functional monomer interactions were thoroughly investigated, ACV–AM interaction energies were calculated, and the optimal ACV/AM ratio was determined with the assistance of computational tools using the density functional theory (DFT) approach. DFT calculations were performed using the B3LYP method and the 6-31G(d,p) basis set.

The combination of MIP and an electrochemical sensor is known to be an effective strategy for electrochemical sensors. This work describes the fabrication of a MIP-based electrochemical sensor directly on a GCE for the highly selective and sensitive determination of ACV. The AM–ACV@MIP/GCE sensor was successfully applied to standard solutions, commercial human serum samples, and tablets to determine ACV. This work, to the best of our knowledge, describes the first MIP-based electrochemical sensor to evaluate ACV in biological and pharmaceutical specimens, exhibiting superior specificity, exceptional sensitivity, stability, and precision by using an electrochemical approach.

## 2 Experimental Section

### 2.1 Reagents and Chemicals

The Soil Products Office in Türkiye gifted the ACV API powder, while the pharmacy provided the Asiviral 400 mg tablets. All reagents, including dopamine (99.0%), ascorbic acid (AA) (≥99.0%), acetic acid (99.0%), uric acid (UA) (≥99.0%), sodium hydroxide (>97.0%), potassium nitrate (≥99.0%), acetaminophen (≥99.0%), acetone (99.5%), magnesium chloride (≥98.0%), methanol (99.9%), sodium sulfate (99.0%), acetonitrile (99.9%), potassium chloride (≥99.0%), human serum, ethylene glycol dimethacrylate (EGDMA) (≥98.0%), HEMA (≥99.0%), 2-hydroxy-2-methylpropiophenone (≥97.0%), potassium ferricyanide (99.0%), and potassium ferrocyanide (≥99.0%) were purchased from Sigma-Aldrich.

Without any form of treatment, the items utilized in the studies were employed right away. Each week, standard stock solutions of 1 mM ACV in 5 mL of methanol, 10.0 mM AM in 5 mL of water, and 5 mM [Fe(CN)_6_]^3–/4–^ (1:1) in 0.1 mM KCl were prepared. These solutions were then sonicated for 10 min in an ultrasonic bath. A refrigerator set to 4 °C was used to keep all solutions once they had been prepared using ultrapure water (with a resistivity of at least 18 MΩ cm at 25 °C).

### 2.2 Equipment/Apparatus

Employing a potentiostat/galvanostat system (AUTOLAB) programmed by the NOVA 2.1.6 software, all electrochemical investigations, including cyclic voltammetry (CV), DPV, and electrochemical impedance spectroscopy (EIS), were performed. An Ag/AgCl ([KCl] = 3 mol/L) reference electrode, a platinum wire auxiliary electrode, and the MIP/nonimprinted polymer (NIP)-altered GCE (diameter = 3.0 mm) as the working electrode were the electrochemical cell components employed for the electrochemical studies. The necessary quantity of chemicals was weighed using a precision balance made by the Ohaus Corporation (Shanghai, China). During the development of the MIP-based sensor, a thermoshaker (Biosan TS-100) was employed in a number of stages. Other devices utilized throughout the investigations included an ultrasonic bath (JP Selecta, Barcelona, Spain) and a vortex mixer (ISOLAB Laborgeräte GmbH, Germany).

Scanning electron microscopy (SEM) (Tescan GAIA3 SEM-FIB, Czech Republic) is employed to examine the surface structure of the films. The Shimadzu IRSpirit-T (Shimadzu, Japan) was utilized to investigate the polymeric film’s infrared spectra using the attenuated total reflectance–Fourier transform infrared spectroscopy (ATR–FTIR) mode. The midinfrared region (4000 to 650 cm^–1^) was used for the scan of the polymeric material.

### 2.3 Preparation of the MIP- and NIP-Based Electrodes with a Polymeric Film

Prior to using the photopolymerization technique, GCE was sonicated in a mixture of double-distilled water and methanol (1:1, v/v) for 15 min, and the surface of the electrode was then polished using alumina slurry. It was then dried at ambient temperature after being rinsed with double-distilled water. ACV (1 mM template molecule, 20 μL), AM solution (10 mM functional monomer, 20 μL), HEMA (99.0% basic monomer, 100 μL), and EGDMA (98.0% cross-linker, 20 μL) were vortexed in an Eppendorf tube to create the photopolymerization mixture. Twenty μL of the mixture was transferred to a new Eppendorf tube, and 2.0 μL of the photoinitiator 2-hydroxy-2-methyl propiophenone (97.0%) was added. The resulting mixture was then vortexed for 1 min. On the surface of the electrode, 0.25 μL of the mixture was transferred, and it was polymerized for three min under a UV lamp (365 nm, 100 W). The electrode was submerged in a glacial acetic acid and methanol mixture (1:1, v/v) for 10 min in a thermoshaker (650 rpm, 25 °C) to remove the template from the framework of the polymer. The electrode was subjected to a particular quantity of ACV (5 × 10^–10^ M) in a thermoshaker (500 rpm, 25 °C) for 25 min to allow the template molecule to rebind. The same working procedure was used to create a NIP-based electrode but without the addition of ACV to measure the efficacy of imprinting. Comparisons were conducted between MIP-based and NIP-based sensors. As a redox probe, a [Fe(CN)_6_]^3–/4–^ mixture was used throughout every electrochemical study, and indirect measurements were performed.

### 2.4 Analysis of ACV in Tablet Dosage Form and Serum Sample Applications

Five ACV tablets, each having 400 mg of ACV, were precisely weighed, thoroughly ground, and blended for this. Tablet powder equivalent to prepare 1 mM ACV solution was weighed and transferred to a 50 mL volumetric flask. It was dissolved in methanol, and the solution was sonicated for 15 min in an ultrasonic bath. After filtering, this solution was utilized to make further experimental solutions. The calibration line obtained by the regression analysis was used to calculate the quantity of ACV. After that, to demonstrate the accuracy of the procedure, recovery tests were conducted after introducing a known quantity of the pure drug substance to the ACV tablet solution.

Commercial human serum (3.6 mL) kept in a deep freezer at −20 °C was mixed with 5.4 mL of acetonitrile and 1.0 mL of an ACV solution (1 mM in methanol) in a 10 mL centrifuge tube in order to determine the applicability in the serum samples. To separate the protein residues from the serum, the solution was centrifuged for 20 min at 5000 rpm. The required dilutions were prepared and used for calibration and recovery tests after the supernatant was collected from the centrifuge tube. From all of the obtained values, five repeatable measurements were used for the calculation of the relative standard deviation (RSD) values.

### 2.5 Computational Methods

All calculations were carried out using Gaussian 09^36^ and GaussView 5^37^ software packages, and Discovery Studio Visualizer^38^ was used in the representation of the results. Geometry optimizations and frequency analyses were performed for ACV, AM, and ACV–AM complexes with ratios of 1:1 to 1:5. In these calculations, the B3LYP method and 6-31G(d,p) basis set were used. In the calculation of binding energy, the equation below is used
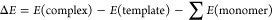
where *E*(complex) is the total energy of the monomer–template complex, *E*(template) is the energy of the template, and ∑*E*(monomer) is the total energy of the functional monomer.^39^

In addition to geometry optimizations and frequency analysis, frontier molecular orbitals and molecular electrostatic potential maps of the investigated compounds and possible ACV–AM complexes were obtained. Molecular electrostatic potential maps are highly useful tools for determining the electron-rich and electron-deficient regions of a given molecule. In this study, molecular electrostatic potential maps were calculated and used to predict how ACV and AM will orient themselves for effective interaction. Thus, it has been determined which groups can act as hydrogen bond donors and which groups can act as hydrogen bond acceptors. Additionally, frontier molecular orbital calculations were performed to determine how template molecules and functional monomers interact and the effects of functional monomers on the template–monomer complex.

## 3 Results and Discussion

### 3.1 Computational Results

Geometry optimizations, molecular electrostatic potential maps, and frontier molecular orbital calculations for the template molecule (ACV) and the functional monomer (AM) were performed, and results are given in Figures S1a–d and S2a–d. The results showed that negative charge is predominantly located on carbonyl oxygens and nitrogen atoms that do not carry hydrogen atoms, while the –OH hydrogen, –NH hydrogen, and –NH_2_ hydrogens acted as positive centers.

**Figure 2:**
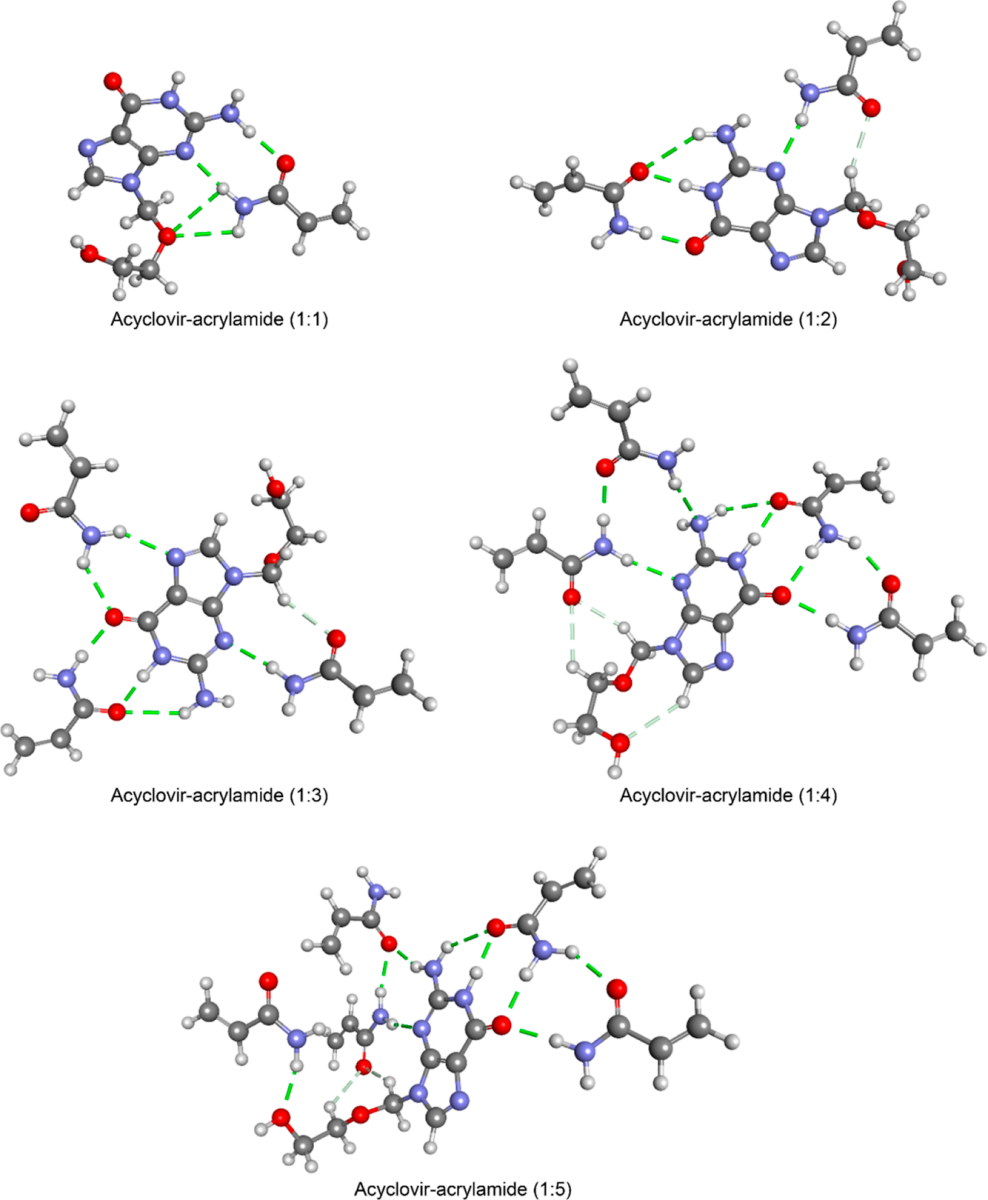
Geometry-optimized structures of ACV–AM complexes and hydrogen bonds formed between ACV and AM.

Geometry optimizations were also carried out on ACV–AM complexes with ratios of 1:1 to 1:5. Calculations were performed on various template–monomer geometrical configurations for each ACV–AM complex (1:1–1:5) to obtain the most stable geometrical configuration. The obtained structures and hydrogen bonds formed between ACV and the functional monomer are given in Figure 2. Results showed that the positive and negative centers that appeared in the molecular electrostatic potential maps took part in the formation of hydrogen bonds (Figures 2 and S3).

The study also performed frontier molecular orbital and energy calculations for template–functional monomer complexes. Results showed that the incorporation of AM into the complex structure results in an increase in the highest occupied molecular orbital (HOMO) energy (from −128.70 to −125.51 kcal/mol) and a decrease in the lowest unoccupied molecular orbital (LUMO) energy (from −3.82 to −23.50 kcal/mol) (Figure S4). Thus, due to a smaller HOMO–LUMO gap, electron transfer can be facilitated, making the detection of the analyte more feasible. Binding energies for each ACV–AM complex were also calculated, and the results are listed in Table 1.

**Table 1:** Binding Energies Were Calculated for Each ACV–AM Complex

ACV/AM mol ratio	*E* (complex) (kJ/mol)	*E* (template) (kJ/mol)	Σ*E* (monomer) (kJ/mol)	Δ*E* (kJ/mol)
1:1	–664055.38	–508857.23	–155182.86	–15.28
1:2	–819246.86	–508857.23	–310364.47	–25.16
1:3	–974438.92	–508857.23	–465547.51	–34.18
1:4	–1129635.52	–508857.23	–620742.52	–35.76
1:5	–1284823.75	–508857.23	–775924.80	–41.71

The results showed that the optimum ACV–AM ratio is 1:1, and this result is also consistent with the experimental results. Increasing the AM ratio leads to better binding of the template molecule ACV to the polymer, hence making it more challenging to remove the template molecule from the MIP surface. Similar results have also been reported for the findings obtained from similar studies in the literature.^40^

### 3.2 Surface Characterization of the Developed Sensor

**Figure 3:**
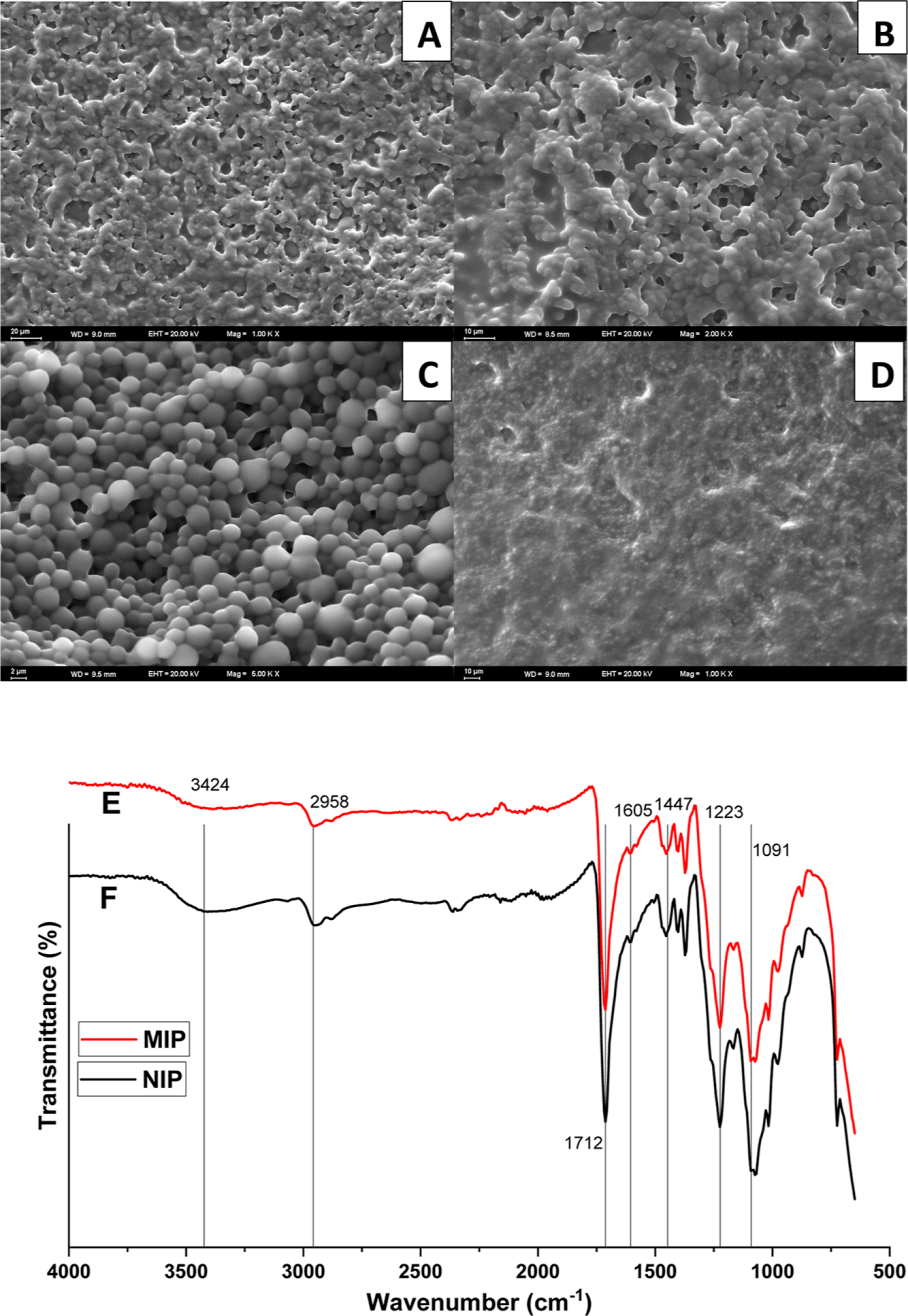
Examination of the surface electrode. SEM images of MIP (A–D) and ATR–FTIR spectra of MIP (E) and NIP (F).

SEM images were used to perform a comprehensive morphological analysis of the sensor surface. The primary emphasis was on two types of polymers: MIPs and NIPs. The goal was to compare the two surfaces’ structural properties and any notable differences. SEM photographs showed a strong distinction between the MIP and NIP surfaces in the AM-ACV@MIP/GCE sensor. To make the comparison easier, these two surfaces are displayed side by side in Figure 3. MIP and NIP surfaces are clearly distinguishable in the SEM pictures acquired for the AM-ACV@MIP/GCE sensor. A rough and porous texture was seen on the MIP’s surface, which was to be expected given the printing technique. These binding sites are designed to capture the target analyte selectively (Figure 3A–C).

Furthermore, improving the porosity and roughness of MIPs is essential for increasing their binding efficiency. The NIP-based surface’s SEM picture is displayed in Figure 3D. A smooth texture with few imperfections was revealed by analysis. This lack of porosity and roughness is in line with what we expected because NIPs lack the MIPs’ typical selective binding sites. Consequently, the results verified that the intended morphological features of MIP- and NIP-based sensor surfaces are present.

Using its ATR–FTIR spectra, the structure of poly(HEMA-AM) was examined to verify the existence of surface functional groups. The FTIR spectra of MIP films are shown in Figure 3E. The corresponding peaks for −CO, −C–O–C, and −C–O vibrations were observed at 1712, 1166, and 1091 cm^–1^, respectively.^41^ While the amide I band resulting from the stretching of the C = O group was obtained at 3424 cm^–1^, the amide II band resulting from –NH_2_ deformation in primary amides was recorded at 1605 cm^–1^. Additionally, amide III stretching vibrations resulting from mixed vibrations of N–H bending and C–N stretching in secondary amides are attributable to the distinct peaks at 1447 and 1223 cm^–1^.^42^ These findings show that functional groups were effectively added to the film’s surface. As can be seen from Figure 3, since both NIP and MIP films contain the same functional monomer AM, the same peaks were observed in the FTIR spectra (Figure 3E,F).

### 3.3 Electrochemical Characterization of the AM-ACV@MIP/GCE Sensor

**Figure 4:**
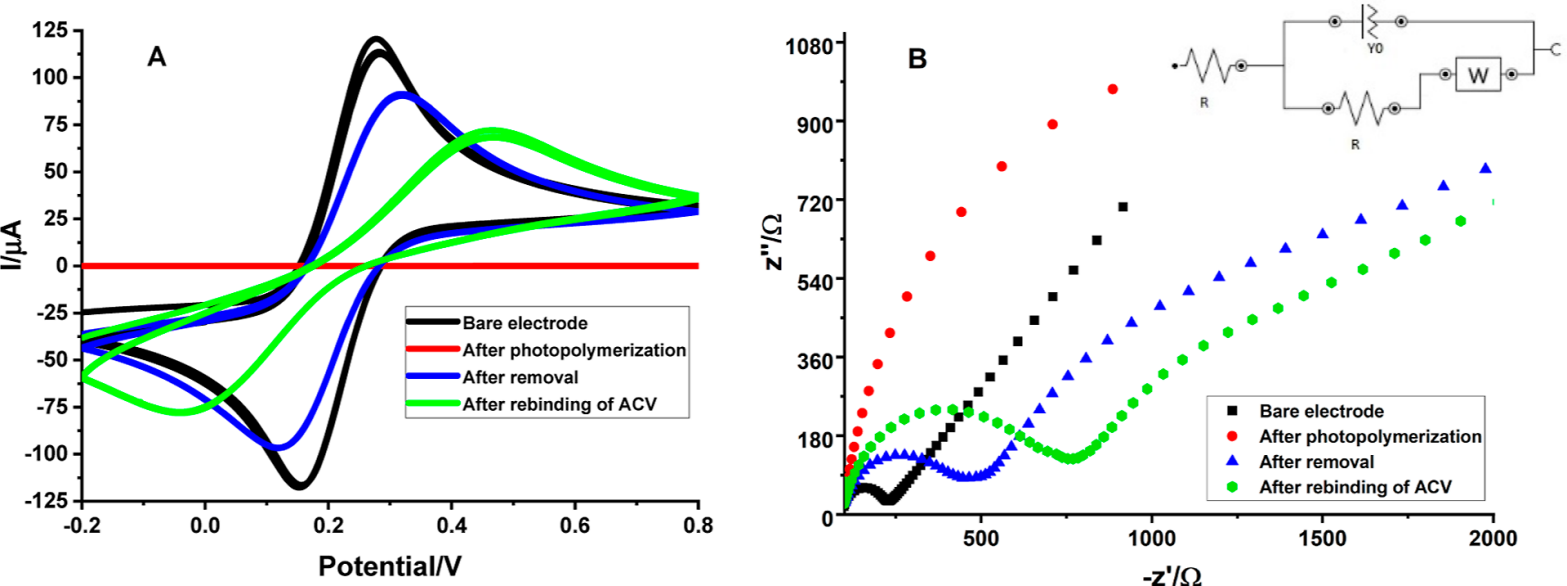
Measurements of GCE’s (A) CV and (B) EIS before and following polymerization, after a template extraction procedure, and after rebinding ACV in 5 mM [Fe(CN)_6_]^3–/4–^ solution (for CV, potential scan range: −0.2 to +0.8 V, scan rate: 0.05 V/s, and step potential: 0.01 V; for EIS, minimum frequency: 0.1 Hz, maximum frequency: 100,000 Hz, and *E*_ac_: 0.01 V).

At various stages following polymerization, removal, and rebinding, in a 5 mM redox probe, [Fe(CN)_6_]^3–/4–^ solution in 0.1 M KCl, CV and EIS tests were carried out to validate the electrochemical characteristics of the AM-ACV@MIP/GCE sensor. After every alteration procedure, as illustrated in Figure 4, the anodic and cathodic peak heights are changed. Due to the absence of any obstructions to electron transport on the empty surface of GCE, the highest peak current levels were observed (Figure 4A). Due to the constraints of electron transport following the photopolymerization technique, smaller anodic/cathodic peak current values of the redox probe have been observed as predicted, demonstrating that effective imprinting was carried out. Unique cavities appeared when the ACV molecule was extracted from the polymeric film, and a redox peak value was enhanced compared with that of the polymerized sensor. Eventually, the peak current value was reduced again after the rebinding process with a particular concentration. The ACV molecules that are attached to some of the cavities on the MIP sensor might provide an explanation for this phenomenon.

Additionally, variations in electrode impedance were assessed by using Nyquist plots from an EIS study and variations in charge transfer resistance (*R*_ct_) (Figure 4B). The EIS findings show that the unmodified GCE surface has the smallest *R*_ct_ value (122 Ω), whereas the polymerization reaction produces the greatest *R*_ct_ value (2300 Ω). Distinct cavities become accessible on the MIP interface after the extraction of ACV molecules, and the *R*_ct_ (323 Ω) value drops as the electron transport becomes more accessible. Even so, as a result of the rebinding of ACV, some of the cavities are occupied, making electron transport challenging, which causes the *R*_ct_ value (569 Ω) to rise once again.

In addition, the Randles–Sevcik equation (*I*_p_ = 2.69 × 10^5^*n*^3/2^*AD*^1/2^ ν ^1/2^ C)^43^ was used to calculate the electroactive surface areas of GCE before polymerization, after polymerization, after removal, and after rebinding. In this equation, *I*_p_ stands for the peak current, *n* stands for the number of transferred electrons (calculated as 1 for potassium ferri/ferrocyanide), *A* stands for the active surface area (cm^2^), *D* stands for the diffusion coefficient (calculated as 7.6 × 10^–6^ cm^2^ s^–1^ for potassium ferri/ferrocyanide), ν stands for the scan rate, and *C* stands for the concentration of probe. The electroactive surface areas of GCE before polymerization were obtained as 0.117 cm^2^, after polymerization as 0.0041 cm^2^, after removal as 0.094 cm^2^, and after rebinding as 0.065 cm^2^. These results can be explained by the coating of the surface after polymerization and the decrease in the active surface area.

### 3.4 Optimization Parameters

#### 3.4.1 Monomer/Template Ratio

**Figure 5:**
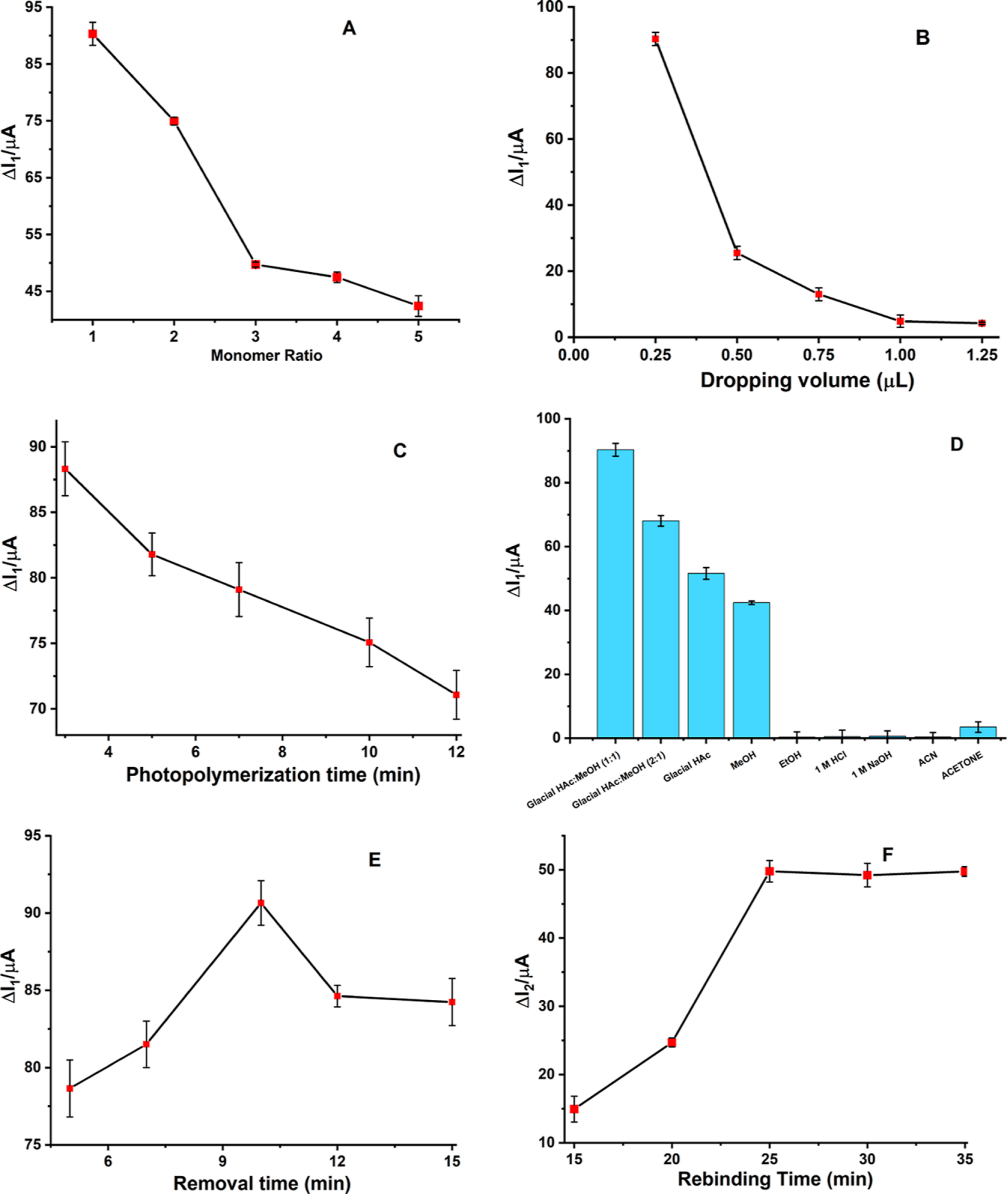
Plots of the Δ*I*_1_ values versus (A) monomer ratio, (B) dropping volume, (C) photopolymerization time, (D) removal solutions, and (E) removal time and Δ*I*_2_ values versus (F) rebinding time. The DPV technique was used to conduct the measurements in a 0.01 mol L^–1^ [Fe(CN)_6_]^3–/4–^ solution. Conditions: potential scan range, −0.2 to +0.8 V; scan rate, 0.001587 V/s; step potential, 8 mV; modulation amplitude, 50 mV; modulation time, 0.05 s; interval time, 0.5 s.

The monomer-to-template ratio is one of the most crucial factors influencing the development of a reliable and efficient polymer since it directly relates to the bonding between the template and the monomer. The most suitable functional monomer was found to ensure that the interactions were specific to the target molecule and to ensure nonspecific interactions with the functional groups present on the target molecule. The different monomers, such as AM, 4-aminobenzoic acid (4-ABA), 4-aminophenol (4-AP), and boronic acid derivatives, were tried. The monomer that gave the best and most stable results was used in the next steps. The variation between peak current values (Δ*I*_1_) recorded after removal and after polymerization is shown in Figure 5A as a function of different molar ratios of the monomer and template (from 1:1 to 5:1). Consequently, the molar ratio of 1:1 was found to be the optimal ratio since it produces the most effective and robust polymer following removal and polymerization. If the functional monomer/template ratio is lower than it should be, functional groups cannot take part effectively in the resulting polymeric structure, and problems occur in the formation of selective cavities. If the ratio is too high, large amounts of functional monomers will be placed irregularly in the polymeric structure, not forming selective cavities. For these reasons, Δ*I*_1_ values were taken into consideration, and the ratio of 1:1, where both effective polymerization and removal, which enable the formation of selective cavities, took place and the Δ*I*_1_ value was the highest, was preferred.

#### 3.4.2 Dropping Volume

Another critical factor is the quantity of the polymerization solution placed onto the GCE surface since it directly impacts the extent and duration of the polymerization. Polymerization cannot occur correctly if the solution’s volume is too big or too small. Five distinct volumes of the polymeric mixture solution (Section 2.3), 0.25, 0.5, 0.75, 1.0, and 1.25 μL, were examined to determine the most suitable volume (Figure 5B). The dropping volume is also directly related to polymeric film thickness and polymerization time. It was observed that Δ*I*_1_ values gradually decreased after 0.25 μL. This can be explained by the fact that the volume on the GCE surface becomes too thick and the removal efficiency decreases. After evaluation of the Δ*I*_1_ values, 0.25 μL was chosen as the ideal volume.

#### 3.4.3 Photopolymerization Time

A UV light with a wavelength of 365 nm was used to prepare the AM-ACV@MIP/GCE sensor. To create a strong and durable polymeric coating on the surface of the GCE, the photopolymerization period under UV light needs to be adjusted. The electrode surface was coated with 0.25 μL of the polymerization mixture and then placed beneath UV light for optimization (3, 5, 7, 10, and 12 min) because, as time increases, the polymeric film formed on the surface may become very thick, making the removal process difficult. The gradual decrease in Δ*I*_1_ values confirms this. Three minutes were found to be the ideal period to build the sensor (Figure 5C).

#### 3.4.4 Removal Solution and Time

The removal of the template molecule during MIP production makes sure that particular cavities are formed for the analyte’s binding. Determining the best removal solution and the time required for removal is therefore crucial. To extract the template from the polymeric layer, several solvents and solutions, including ethanol, glacial acetic acid, methanol, and glacial acetic acid/methanol mixture (1:1 and 2:1 v/v), 1 M hydrochloric acid, 1 M sodium hydroxide, acetone, and acetonitrile, were investigated. Glacial acetic acid and methanol (1:1 v/v) were selected as the removal solution after all the chemicals’ Δ*I*_1_ values were examined (Figure 5D). By subtracting the after-removal and polymerization peak current values, we calculated the Δ*I*_1_ of the removal solvents. The next step is calculating the necessary removal time after choosing the removal solution. Removal times ranging from 5 to 15 min were employed to determine the ideal removal time utilizing the thermoshaker. Figure 5E shows that the peak value was at its maximum at 10 min. After that, it began to decline due to the breakage of polymeric chains and the reconstruction of interpolymeric connections, which caused the imprinted cavities to deform. In addition, the extended incubation period may result in variations in the surface coverage level or the positioning of pores on the polymeric surface. Therefore, 10 min was chosen as the optimum value for further research.

#### 3.4.5 Rebinding Time

The analytical capacities and validation of the generated sensor are affected directly by the template’s rebinding procedure. To ensure strong and steady attachment to the particular pores that are revealed after removal, several incubation times between 15 and 35 min at a concentration of 5 × 10^–10^ M ACV were investigated, as shown in Figure 5F. When comparing the after-rebinding and after-removal peak current difference (Δ*I*_2_), it is evident that Δ*I*_2_ practically remains constant after reaching its maximum value at 25 min. In order to guarantee a robust and effective binding, 25 min was selected as the rebinding duration. Although the optimal rebinding time of 25 min was long, it did not negatively affect the performance of the sensor. The prepared sensor surface was tested by using different rebinding solutions on different days. This showed that the prepared sensor achieved stable and reproducible results despite the long rebinding time.

### 3.5 Analytical Validation of the AM-ACV@MIP/GCE Sensor

**Figure 6:**
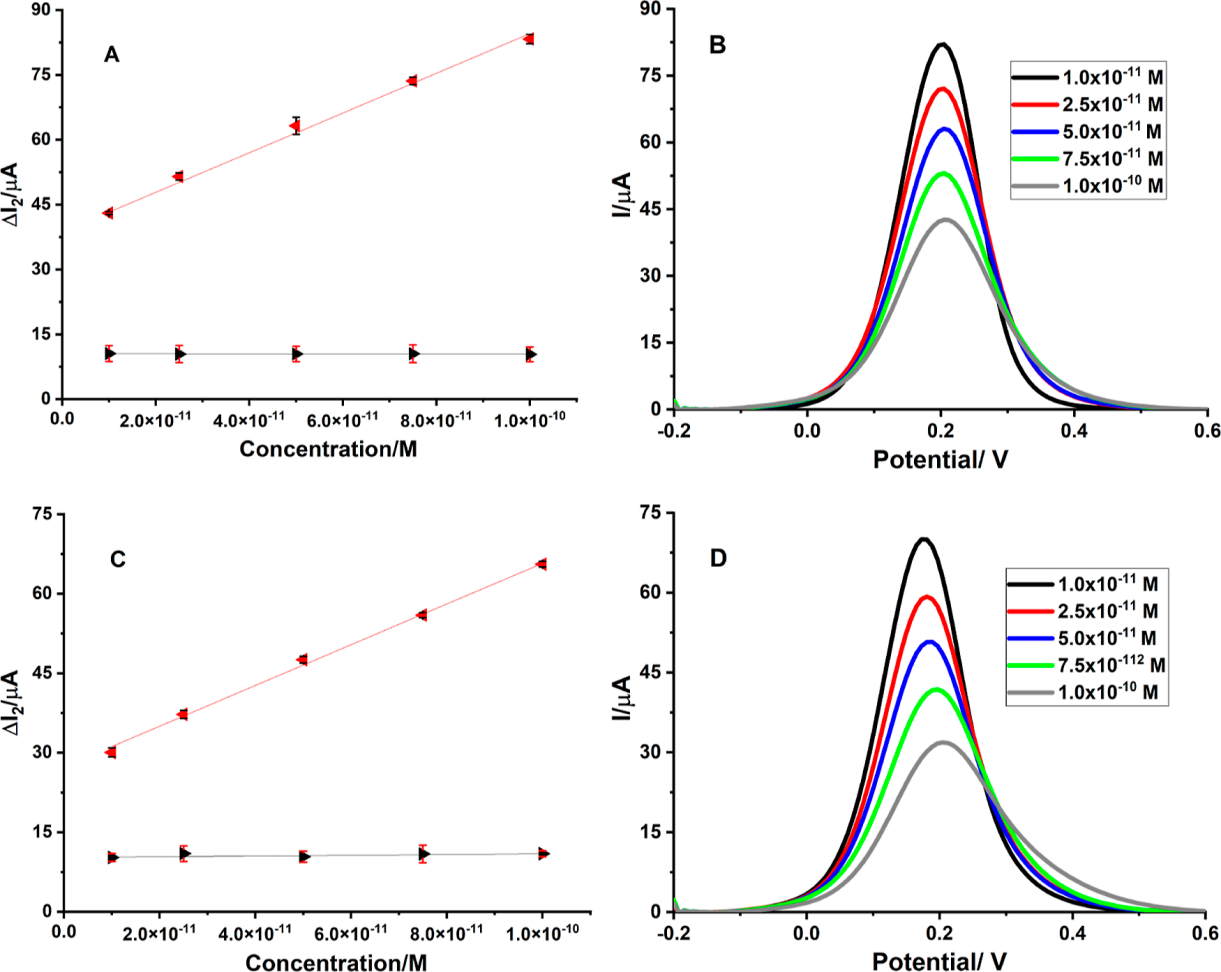
Calibration lines of ACV using sensors based on MIP and NIP in solutions of (A) standard solution and (C) spiked serum sample; DPV voltammograms produced by rebinding various ACV quantities in solutions of (B) standard and (D) spiked serum. The experiments were carried out in 5 mM [Fe(CN)_6_]^3–/4–^ solution (conditions: for DPV; potential scan range, −0.2 to +0.8 V; scan rate, 0.001587 V s ^–1^; step potential, 8 mV; modulation amplitude, 50 mV; modulation time, 0.05 s; and interval time, 0.5 s).

A redox probe [Fe(CN)_6_]^3–/4–^ was used to test the electrochemical behavior and analytical capabilities of the AM-ACV@MIP/GCE sensor. Under the appropriate circumstances, DPV was used to determine ACV via an indirect method (Figure 6A). In the indirect method, the basic principle is that instead of performing electrochemical measurements directly in the solution of the target drug, a redox probe ([Fe(CN)_6_]^3–/4–^) is used. With the DPV measurements, changes in the peak current of the redox probe are examined. After the target molecule, ACV, binds to specific cavities in the MIP, there will be a decrease in the peak current of the redox probe due to the closure of the cavities. This decrease will increase according to increasing concentrations of ACV. In the calibration chart, Δ*I*_2_ values are plotted against concentrations. In this context, determination is made indirectly by calculating the Δ*I*_2_ values. A linear response between 1 × 10^–11^ M and 1 × 10^–10^ M was seen when Δ*I*_2_ values were plotted against the various ACV concentrations (Figure 6A). This calibration graph’s regression equation was Δ*I*_2_ (μA) = 4.58 × 10^11^ C (*M*) + 38.67 (*R*^2^ = 0.998) (Table 2). For the defined range of concentrations, regression data were used to compute the limit of detection (LOD) (LOD = 3 standard deviation/slope) and limit of quantification (LOQ) (LOQ = 10 standard deviation/slope) values, which were 7.15 × 10^–13^ M and 2.38 × 10^–12^ M, respectively.^44^ The calibration lines for these two sensors differ significantly from one another. The MIP curve (represented by the red line) demonstrated a linear response with a proportionate rise in Δ*I*_2_ in relation to the ACV concentration. In contrast, the black line represents the NIP curve, which had a negligible Δ*I*_2_ value in comparison to the MIP because the NIP lacked any distinctive cavities for the ACV. It is evident from this that the AM-ACV@MIP/GCE sensor demonstrated outstanding sensitivity as well as specificity for the ACV measurement.

**Table 2:** Regression Analysis for the ACV Calibration Curve on AM-ACV@MIP/GCE

	standard solution	commercial serum sample
linearity range (M)	1 × 10^–11^ to 1 × 10^–10^	1 × 10^–11^ to 1 × 10^–10^
slope (μA M^–1^)	4.58 × 10^11^	3.84 × 10^11^
SE of slope	9.66 × 10^9^	8.85 × 10^9^
intercept (μA)	38.67	27.34
SE of intercept	0.35	0.63
correlation coefficient (*R*)	0.998	0.998
adjusted *R*^2^	0.992	0.995
residual sum of squares	12.78	6.93
LOD (M)	7.15 × 10^–13^	1.92 × 10^–12^
LOQ (M)	2.38 × 10^–12^	6.40 × 10^–12^
repeatability of response (RSD %)^a^	0.31	0.83
reproducibility of response (RSD %)^a^	1.95	2.54
calculated *t*_value_^a^	0.126	0.387
confidence interval (95%)	±0.199	±0.407

a Each value is the mean of five experiments. The theoretical student-*t* value is 2.13.

### 3.6 Application of the AM-ACV@MIP/GCE Sensor in the Pharmaceutical Dosage Form and Biological Sample

The AM-ACV@MIP/GCE sensor has been effectively used to determine ACV in tablet dosage form and spiked human serum with a high level of accuracy. ACV concentrations between 1 × 10^–11^ M and 1 × 10^–10^ M were used to determine its presence in the spiked serum samples. In the above range, the peak current values showed linearity with the regression equation of Δ*I*_2_ (μA) = 3.84 × 10^11^ C (*M*) + 27.34 (*R*^2^ = 0.998) (Figure 6C). The sensor’s efficiency was demonstrated by the calculation of extremely low LOD and LOQ values (1.92 × 10^–12^ M and 6.40 × 10^–12^ M, respectively). Table 2 provides a summary of the obtained regression data. Figure 6A,C, respectively, illustrate the calibration lines for ACV with AM-ACV@MIP/GCE and the NIP sensors in standard preparation and spiked serum. The AM-ACV@MIP/GCE and NIP sensors comparison figures showed that the current level for the MIP-based sensor is steadily increasing, while it nearly stays unaltered for the NIP-based sensor. The recovery study confirmed the AM-ACV@MIP/GCE sensor’s accuracy. Additionally, the recovery study of the pharmaceutical tablets (recovery % is 99.40%) was used to show the efficacy and practicability of the AM-ACV@MIP/GCE sensor (Table 3).

**Table 3:** Results of the Recovery Studies for the Tablet Dosage Form and Commercial Serum Sample

	tablet dosage form (Asiviral)	commercial serum sample
label amount (mg)	400.00	
found amount (mg)^a^	401.60	
RSD %^a^	0.31	
bias %	+0.40	
spiked amount (mg)	10.000	10.000
found amount (mg)^a^	9.940	10.044
average recovery (%)	99.40	100.44
RSD % of recovery^a^	0.73	0.41
bias %	–0.60	+0.44

a Each value is the mean of five experiments.

### 3.7 Selectivity

Obtaining excellent selectivity compared to that of the template molecule’s analogues is one of the most crucial goals of MIP. Utilizing competitor molecules comparable in structure and chemistry, the selectivity study was conducted. Investigations were carried out on the AM-ACV@MIP/GCE sensor’s selective response to ACV and model medications such as valganciclovir hydrochloride, tenofovir diphosphate, abacavir sulfate, zalcitabine, and lamivudine. The binding specificity of the AM-ACV@MIP/GCE sensor for ACV served as the basis for the evaluation of the selectivity (*k*) and relative selectivity coefficient (*k*′) values (Table 4). The following equations were used to calculate the *k* and *k*′ values
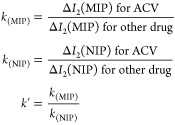


**Table 4:** Calculations of *k* and *k*′ for ACV and Other Structurally Similar Drugs

	AM-ACV@MIP/GCE		NIP		
	ΔI2/μA	*k*_(MIP)_	ΔI2/μA	*k*_(NIP)_	*K*′(MIP/NIP)
ACV	63.22		10.46		
valganciclovir hydrochloride	21.71	2.912	13.77	0.760	3.834
tenofovir diphosphate	22.06	2.866	16.83	0.622	4.611
abacavir sulfate	19.43	3.254	18.83	0.555	5.857
zalcitabine	22.26	2.840	16.36	0.639	4.442
lamivudine	20.52	3.081	16.54	0.632	4.872

**Figure 7:**
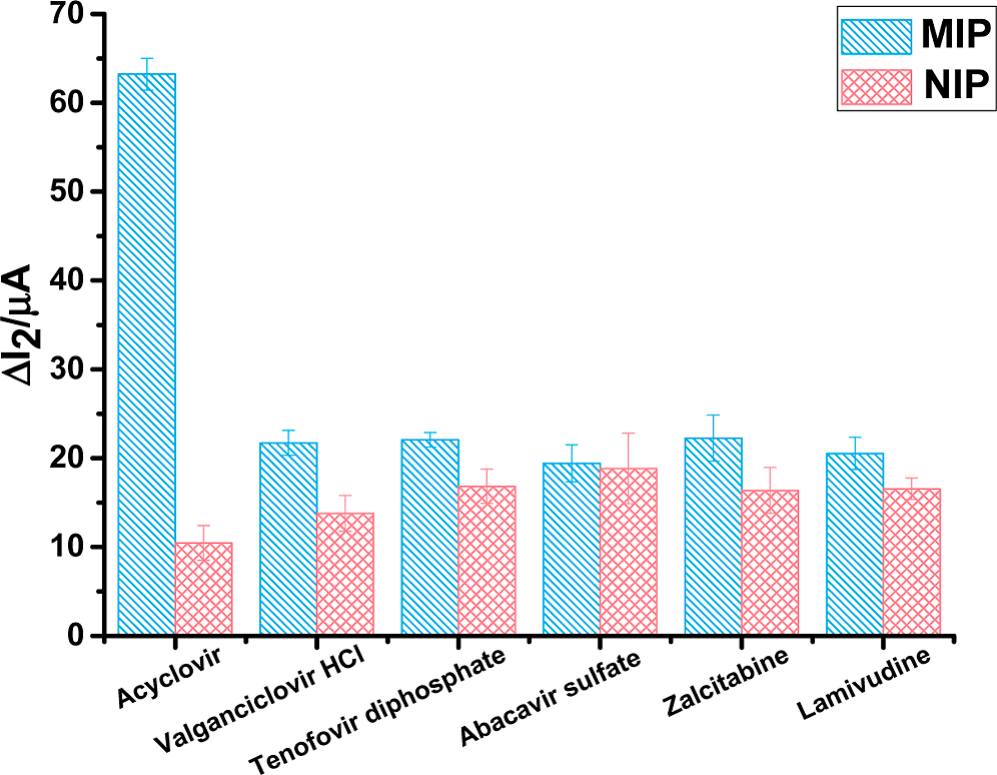
Selectivity of ACV, valganciclovir hydrochloride, tenofovir diphosphate, abacavir sulfate, zalcitabine, and lamivudine corresponding to AM-ACV@MIP/GCE and AM-ACV@NIP/GCE sensors.

Compared to other model drugs, the developed MIP sensor seemed to have superior selectivity in targeting ACV. Furthermore, this showed that ACV specifically rebinds to the electrode’s recognition regions based on the size and shape of the template molecule. As opposed to this, weak noncovalent associations among functional monomers and model drug molecules were responsible for the nonspecific binding to NIP (Figure 7).

### 3.8 Interference Studies

**Figure 8:**
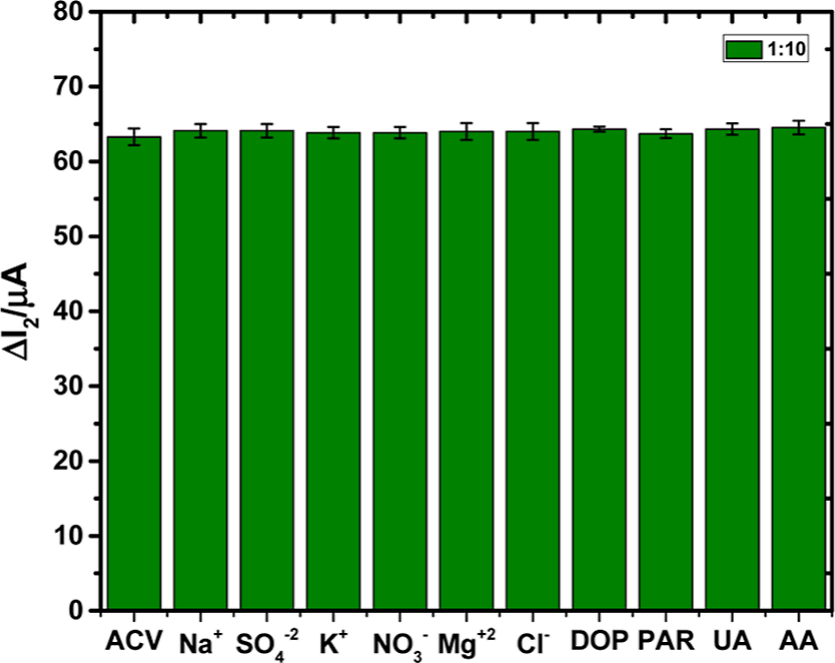
Bar graphs of 5 × 10^–11^ M ACV at the AM-ACV@MIP/GCE sensor.

ACV was examined at a concentration of 5 × 10^–11^ M against probable interfering substances such as K^+^, NO_3_^–^, Na^+^, SO_4_^2–^, Mg^2+^, Cl^–^, dopamine (DOP), paracetamol (PAR), UA, and AA, in addition to selectivity experiments against structurally related competitors. Even though the interfering agents’ molar concentration in this investigation was ten times higher than the concentration of ACV, the peak current level of ACV was not affected considerably. The recovery was determined to be between 100.68 and 101.66%, and the RSD ranged from 0.35 to 1.12%, as shown in Table 5. The findings showed that the AM-ACV@MIP/GCE sensor’s analytical capabilities are unaffected by interfering agents (Figure 8).

**Table 5:** Impact of Different Interferents on the Detection of ACV

interferent	recovery of ACV (%)	RSD (%)^a^
K^+^	100.89	0.77
Cl^–^	101.14	1.12
Na^+^	101.30	0.90
NO_3_^–^	100.89	0.77
Mg^2+^	101.14	1.12
SO_4_^2–^	101.30	0.90
dopamine	101.65	0.35
paracetamol	100.68	0.58
uric acid	101.66	0.77
ascorbic acid	101.10	0.92

a Each value is the mean of three experiments.

### 3.9 Stability

The AM-ACV@MIP/GCE sensor’s stability was assessed over 14 days. Stability was observed by measuring how the sensor response changed during this time interval. On the fifth day, the sensor’s response was 91% of the initial response, and it dropped below 90% after the fifth day. According to these results, it was seen that the AM-ACV@MIP/GCE sensor can be used effectively until the fifth day.

### 3.10 Comparison with Other Methods

Following a thorough review of the scientific literature, many methods that have been effectively developed to determine the ACV were found. Spectroscopic and chromatographic techniques require lengthy procedures for sample preparation, expensive equipment setup, and specialized staff. Even though electrochemical techniques are more straightforward, they frequently fall short of providing the necessary selectivity for detecting drugs in biological samples. In terms of the LOD, linear range, and recovery outcomes, it is evident that the created technique performs significantly better than these approaches. Additional benefits of the created sensor over alternative approaches include superior selectivity, quick analysis times, affordability, and ecological friendliness. For a comparative investigation of actual samples, the AM-ACV@MIP/GCE sensor demonstrated accurate, fast, and easy detection. Table 6 presents a summary of the different ACV assay techniques.

**Table 6:** Comparing Analytical Methods Created for the Determination of ACV

methodology	linear range	LOD	sample	recovery (%)	references
UV/vis	10–30 μg/mL	0.3 μg/mL	dosage forms	96.9–102.0	(45)
UV/vis	1.81–9.06 μg/mL	0.024 μg/mL	dosage forms	99.94–101.43	(46)
HPLC	8–12 μg/mL	0.27 μg/mL	dosage forms	99.56–100.61	(47)
RP-HPLC	25.0–150.0 ng/mL	8 ng/mL	human plasma	96.98–98.91	(48)
UHPLC-MS/MS	0.05–50 mg/L	0.05 mg/L	human serum	92.2–114.2	(49)
SWV	10 nM–30 μM	1.8 nM	dosage forms, human urine	96.0–102.7	(50)
SWV	5.0 × 10^–8^–8.0 × 10^–7^ M	1.55 nM	dosage forms, human urine	98.8–100.7	(16)
DPV	0.01–118 and 148–918 μM	0.02 μM	dosage forms, human urine	88–96.6	(51)
DPV	0.03–0.3 and 0.3–1.5 μM	12 nM	dosage forms, human serum	98–102%	(25)
MIP-HPLC	0.10–50.0 μg g–^1^	0.09 μg g–^1^	creatural tissue	85.5–108.1	(52)
MIP-HPLC	10–400 ng/mL	1.8 ng/mL–^1^	human serum	95.6	(53)
AM-ACV@MIP/GCE	1 × 10^–11^–1 × 10^–10^ M	7.15 × 10^–13^ M	dosage forms, human serum	99.40 and 100.44	this study

## 4 Conclusions

This work offers a generic procedure for making MIP-based, high-performance electrochemical sensors. In contrast to previously published analytical methods, this work develops an electrochemical MIP-based sensor that is straightforward, inexpensive, extremely sensitive, and specific for the first attempt to determine ACV. The designed sensor was produced by the photopolymerization technique employing the AM monomer. The electrochemical behavior of the MIP-based sensor was validated by using CV and EIS methods. The linear working range and LOD were estimated as 1 × 10^–11^ to 1 × 10^–10^ M and 7.15 × 10^–13^ M, respectively. The acquired recovery values have been observed to be 100.44 and 99.40%, respectively, when it was applied to commercial serum and tablet dosage forms. The excellent selectivity of the AM-ACV@MIP/GCE sensor allowed for accurate measurement of ACV in human plasma and pharmaceutical forms. Additionally, the suggested sensor is easy to manufacture and handle, reliable, sensitive, exceptionally selective, and economical. Altogether, this study presents a novel avenue for the development of effective MIP sensors and offers fresh approaches to the fast and accurate determination of ACV. AM-ACV@MIP/GCE can therefore be considered a desirable analytical instrument for point-of-care diagnostic tests and quality control in the pharmaceutical sector. Future research potential to construct point-of-care devices for various pharmaceuticals could be rendered possible by this sensor’s performance. The use and advancement of MIP sensors for the identification of various drug compounds from pharmaceutical preparations, biological samples, and environmental samples are anticipated to be encouraged in the future by this research. Integration of MIP-based electrochemical sensors with more versatile and portable options, especially screen-printed electrodes, may enable MIP sensors to find use in routine drug analysis. Obtaining sensors with high stability will also assist in this use.

## Supporting Information Available

Two-dimensional structure, geometry-optimized structure, molecular electrostatic potential map, frontier molecular orbitals, and HOMO and LUMO energies of ACV and AM; molecular electrostatic potential maps of template–monomer complexes; and effect of the functional monomer on frontier molecular orbitals and the HOMO–LUMO gap (PDF)
