# Supplementary material for: Development and Fabrication of a Molecularly Imprinted Polymer-Based Electroanalytical Sensor for the Determination of Acyclovir
Source: ACS Omega. 2024 Feb 12;9(8):9564–76. doi: 10.1021/acsomega.3c09399 (PMC10905707; doi:10.1021/acsomega.3c09399)
Supplement: Supplementary file 1 — ao3c09399_si_001.pdf [file ao3c09399_si_001.pdf]

Supplementary Material

Development and Fabrication of a Molecularly Imprinted Polymer-Based Electroanalytical Sensor for the Determination of Acyclovir

Abdullah Al Faysal[a], Ahmet Cetinkaya[b c], S. Irem Kaya[d], Taner Erdoğan[e], Sibel A. Ozkan[b*], Ayşegül Gölcü[a*]

[a]Department of Chemistry, Faculty of Sciences and Letters, Istanbul Technical University, Maslak, Istanbul, Turkey

[b]Faculty of Pharmacy, Department of Analytical Chemistry, Ankara University, 06560, Turkey

[c]Ankara University, Graduate School of Health Sciences, 06110, Turkey

[d]University of Health Sciences, Gulhane Faculty of Pharmacy, Department of Analytical Chemistry, Ankara, Turkey

[e]Kocaeli University, Kocaeli Vocational School, Department of Chemistry and Chemical Processing Technologies, Kocaeli, 41140, Turkey.

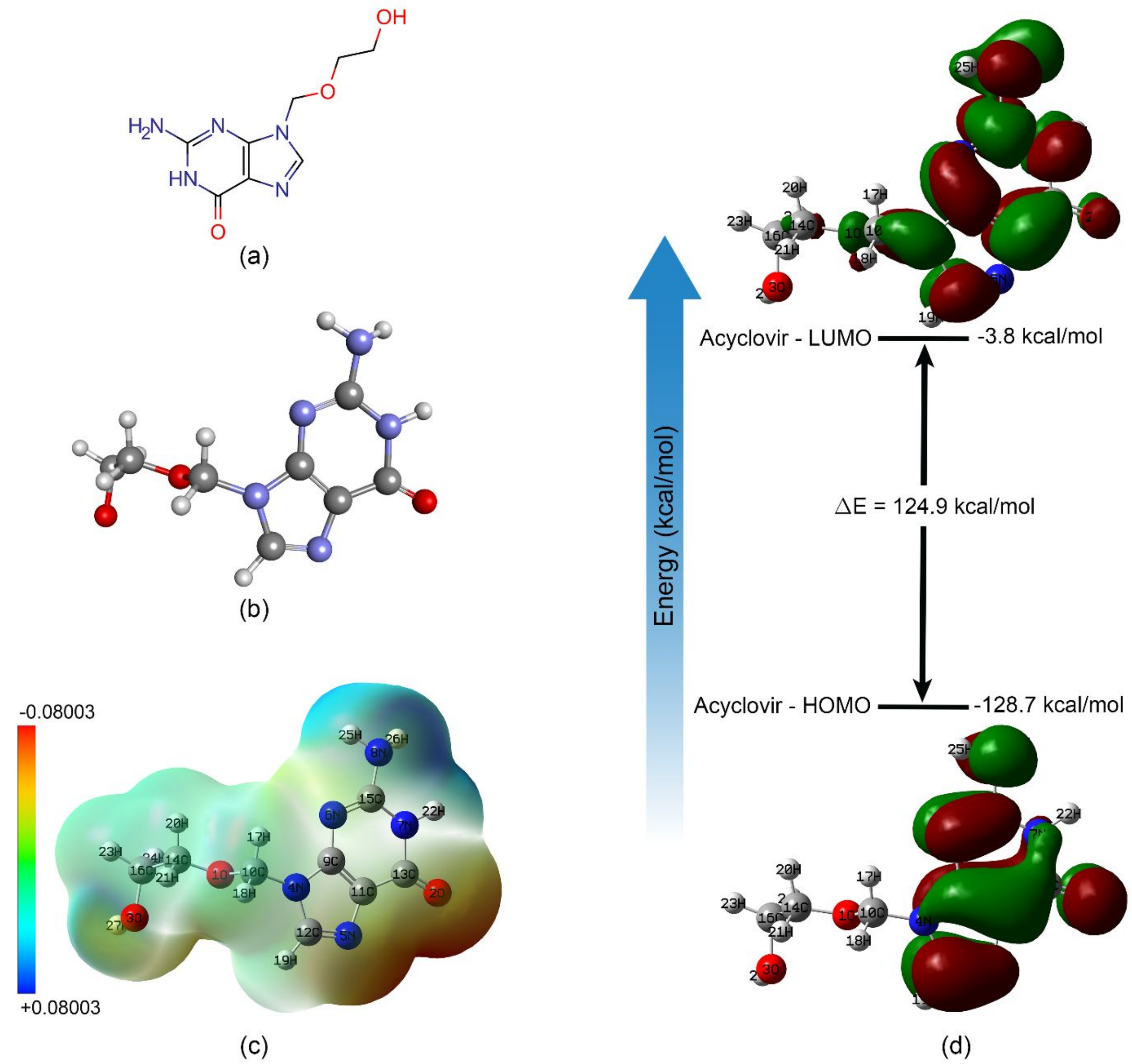

Figure S1. (a) 2D structure, (b) geometry optimized structure, (c) molecular electrostatic potential map and (d) frontier molecular orbitals and HOMO, LUMO energies of acyclovir.

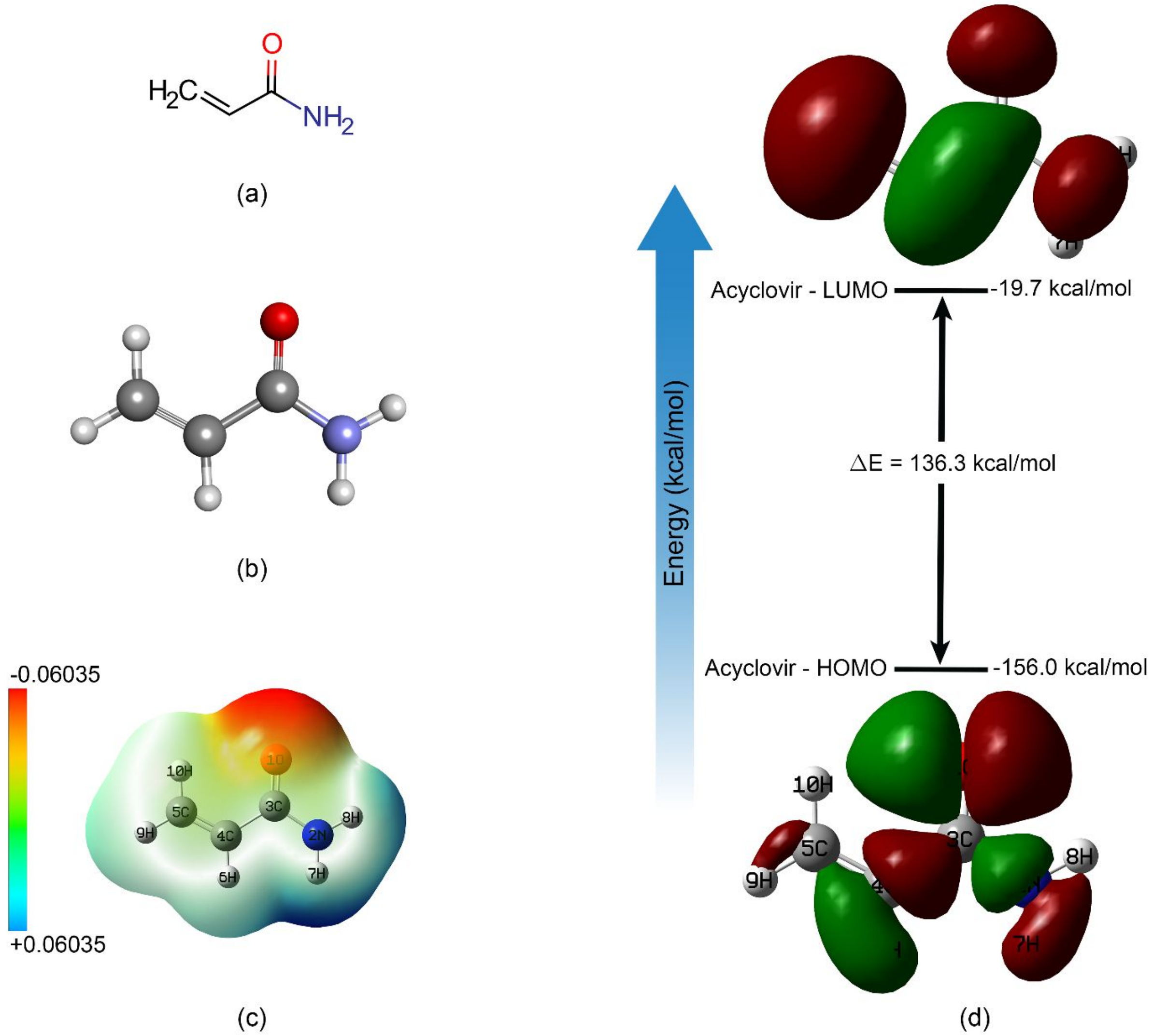

Figure S2. (a) 2D structure, (b) geometry optimized structure, (c) molecular electrostatic potential map and (d) frontier molecular orbitals and HOMO, LUMO energies of acrylamide.

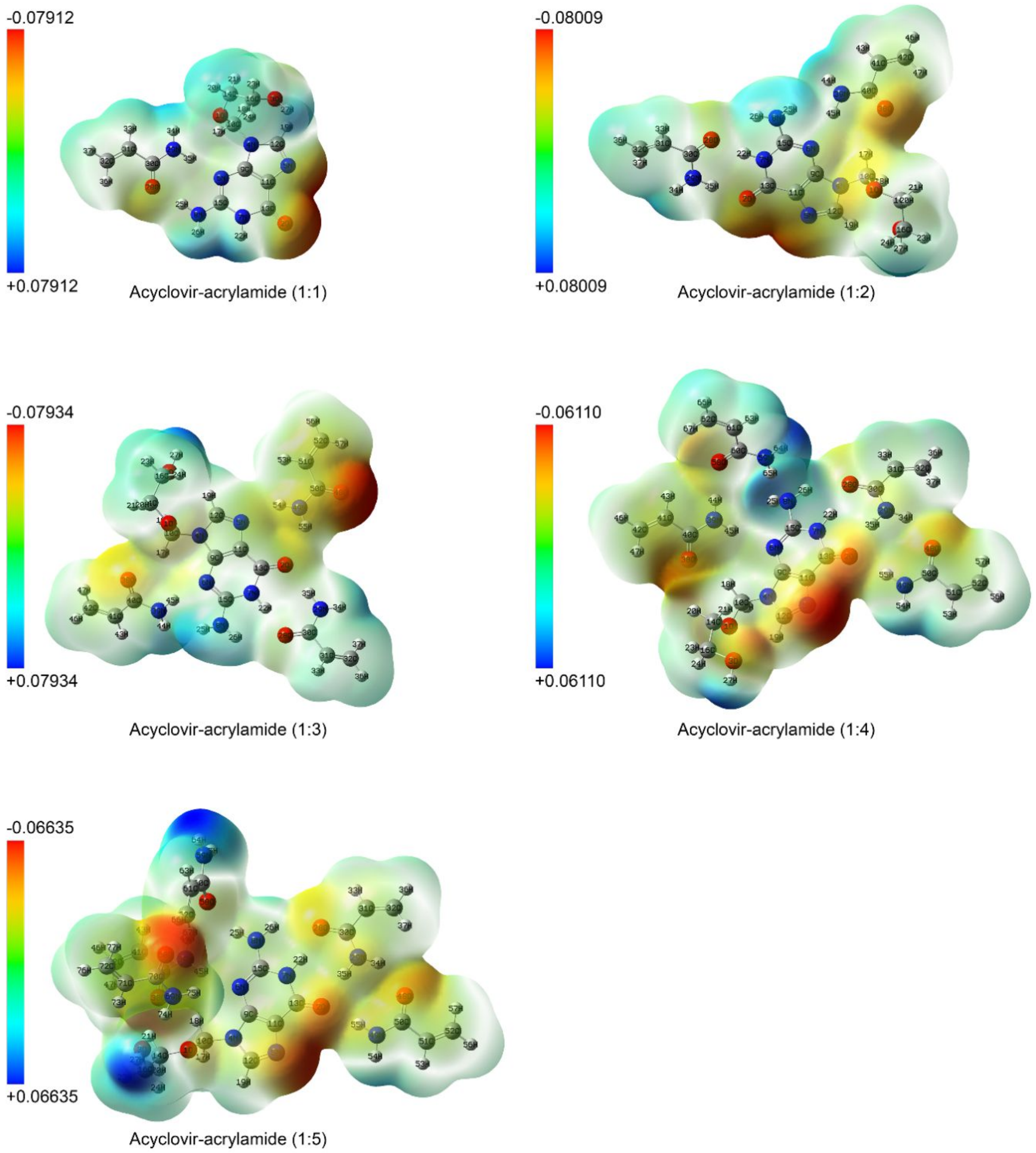

Figure S3. Molecular electrostatic potential maps of template-monomer complexes.

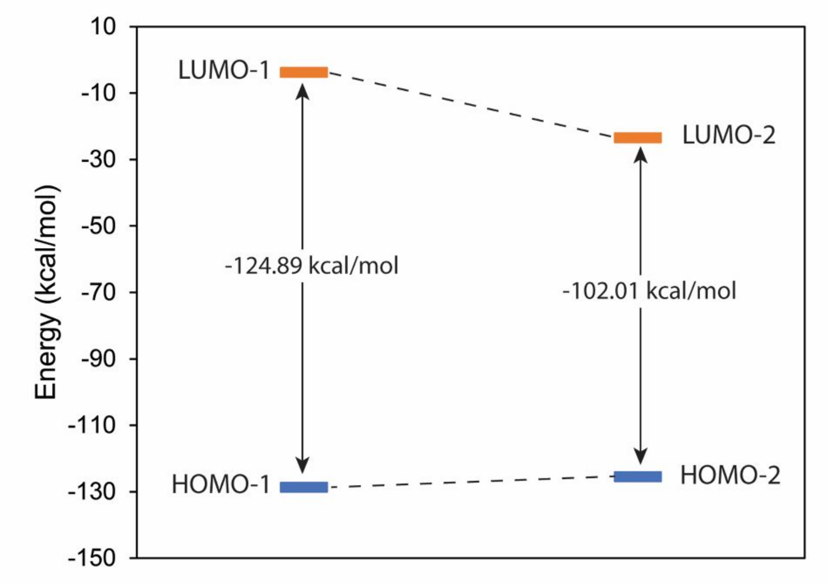

Figure S4. Effect of functional monomer on frontier molecular orbitals and HOMO-LUMO gap.
